# Role of ICAM-1 in the Adhesion of T Cells to Enteric Glia: Perspectives in the Formation of Plexitis in Crohn’s Disease

**DOI:** 10.1016/j.jcmgh.2024.02.016

**Published:** 2024-02-29

**Authors:** Julie Pabois, Tony Durand, Catherine Le Berre, Rhiannon T. Filippone, Théo Noël, Emilie Durieu, Céline Bossard, Sarah Bruneau, Malvyne Rolli-Derkinderen, Kulmira Nurgali, Michel Neunlist, Arnaud Bourreille, Isabelle Neveu, Philippe Naveilhan

**Affiliations:** 1 Nantes Université, CHU Nantes, Inserm, TENS, The Enteric Nervous System in Gut and Brain Diseases, IMAD, Nantes, France; 2 Service d’Anatomie et Cytologie Pathologique, Inserm, CRCINA, Université de Nantes, CHU Nantes, Nantes, France; 3 CHU Nantes, Nantes Université, INSERM, Center for Research in Transplantation and Translational Immunology, UMR 1064, Nantes, France; 4 Institute for Health and Sport, Victoria University, Melbourne, VIC, Australia; 5 Department of Medicine Western Health, The University of Melbourne, Melbourne, Australia; 6 Regenerative Medicine and Stem Cell Program, Australian Institute for Musculoskeletal Science (AIMSS), Melbourne, Australia

**Keywords:** Plexitis, Chronic Colitis Mouse Model, Lifitegrast, Glial Cells

## Abstract

**Background & Aims:**

The presence of myenteric plexitis in the proximal resection margins is a predictive factor of early postoperative recurrence in Crohn’s disease. To decipher the mechanisms leading to their formation, T-cell interactions with enteric neural cells were studied in vitro and in vivo.

**Methods:**

T cells close to myenteric neural cells were retrospectively quantified in ileocolonic resections from 9 control subjects with cancer and 20 patients with Crohn’s disease. The mechanisms involved in T-cell adhesion were then investigated in co-cultures of T lymphocytes with enteric glial cells (glia). Finally, the implication of adhesion molecules in the development of plexitis and colitis was studied in vitro but also in vivo in Winnie mice.

**Results:**

The mean number of T cells close to glia, but not neurons, was significantly higher in the myenteric ganglia of relapsing patients with Crohn’s disease (2.42 ± 0.5) as compared with controls (0.36 ± 0.08, *P* = .0007). Co-culture experiments showed that exposure to proinflammatory cytokines enhanced T-cell adhesion to glia and increased intercellular adhesion molecule-1 (ICAM-1) expression in glia. We next demonstrated that T-cell adhesion to glia was inhibited by an anti–ICAM-1 antibody. Finally, using the Winnie mouse model of colitis, we showed that the blockage of ICAM-1/lymphocyte function-associated antigen-1 (LFA-1) with lifitegrast reduced colitis severity and decreased T-cell infiltration in the myenteric plexus.

**Conclusions:**

Our present work argues for a role of glia–T-cell interaction in the development of myenteric plexitis through the adhesion molecules ICAM-1/LFA-1 and suggests that deciphering the functional consequences of glia–T-cell interaction is important to understand the mechanisms implicated in the development and recurrence of Crohn’s disease.

## Graphical abstract

**Figure:**
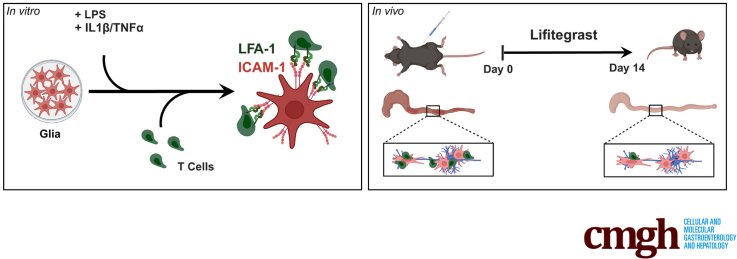

**Summary**

Immune infiltration within enteric neural ganglia is predictive of early postoperative recurrence of Crohn’s disease. Interactions of T lymphocytes with enteric glial cells through ICAM-1/LFA-1 contribute to the formation of plexitis in the gastrointestinal tract.

Crohn’s disease is an inflammatory bowel disease (IBD) that can occur throughout the entire gastrointestinal tract but commonly affects the small bowel and may cause transmural lesions. This destructive and disabling disease leads to progressive and cumulative bowel damage, encompassing strictures, fistulas, and/or abscesses.^1^ Twenty years after diagnosis, more than half of patients experience penetrating or stricturing complications.^2^ Despite the advent of biologics, about 50% of patients still require surgery within 10 years of diagnosis because of those complications, leading to inherent additional bowel damage, and postoperative recurrence is frequent.^3^ Therefore, better identification of the mechanisms and factors contributing to IBD and postoperative recurrence remains a major unmet clinical need.

Abnormal accumulation of immune cells in and around the enteric nervous ganglia, called enteric plexitis, has been observed in the proximal margin of intestinal resections from Crohn’s patients. These plexitis have been determined as a predictive factor for early postoperative recurrence of Crohn’s disease,^4^ and two recent meta-analyses confirmed that the presence of plexitis should be recorded at the time of index resection because of their association with postoperative recurrence.^5^^,^^6^ In light of these observations, the detection of myenteric plexitis for predicting the risk of postoperative recurrence has been incorporated in the guidelines of the European Crohn and Colitis Organization in 2017.

These findings suggest critical crosstalk between the immune system and the enteric nervous system (ENS) that may contribute positively or negatively to the evolution of IBD and its associated dysfunctions. This hypothesis is supported by recent studies reporting that neuroimmune interactions contribute to the control of intestinal homeostasis and inflammation. Indeed, glial cell line-derived neurotrophic factor production by enteric glial cells (glia) in response to toll-like receptor activation decreases the susceptibility of mice to colitis through the induction of interleukin (IL)-22 by the type 3 innate lymphoid cells.^7^

Plexitis has been observed in other inflammatory or functional digestive disorders, including diverticular disease^8^ and idiopathic achalasia.^9^^,^^10^ We recently showed that control patients operated for cancer also had plexitis at the proximal margin of ileocolonic resection, but a higher number of T lymphocytes in proximity to glia was observed in the myenteric ganglia of Crohn’s patients.^11^ Thus, we hypothesize that the nature and/or the density of the inflammatory infiltrate in contact with the ENS, rather than the presence of plexitis per se, would favor the emergence of digestive symptoms, all syndromes combined, and contribute to disease recurrence after surgical resection in the case of Crohn’s disease.

Previous studies reported the presence of lymphocytes, plasmocytes, neutrophils, and eosinophils in enteric plexitis,^12^ but nothing is known about the cell interactions that preferentially lead to their formation and to the development of digestive symptoms and disease recurrence. The present study aimed at deciphering the mechanisms of plexitis formation. The first aim was to characterize the myenteric ganglia of Crohn’s patients and the potential differences between recurrent, non-recurrent, and control patients. The second goal was to define the molecules implicated in the interactions between glia and T cells. The third objective was to examine the involvement of the intercellular adhesion molecule-1 (ICAM-1) and lymphocyte function-associated antigen-1 (LFA-1) in the formation of plexitis in vivo using the Winnie mouse model of spontaneous chronic colitis.

## Results

### The Number of T Cells Interacting With Glia Is Higher in the Myenteric Ganglia of Patients With Crohn’s Disease

**Figure 1:**
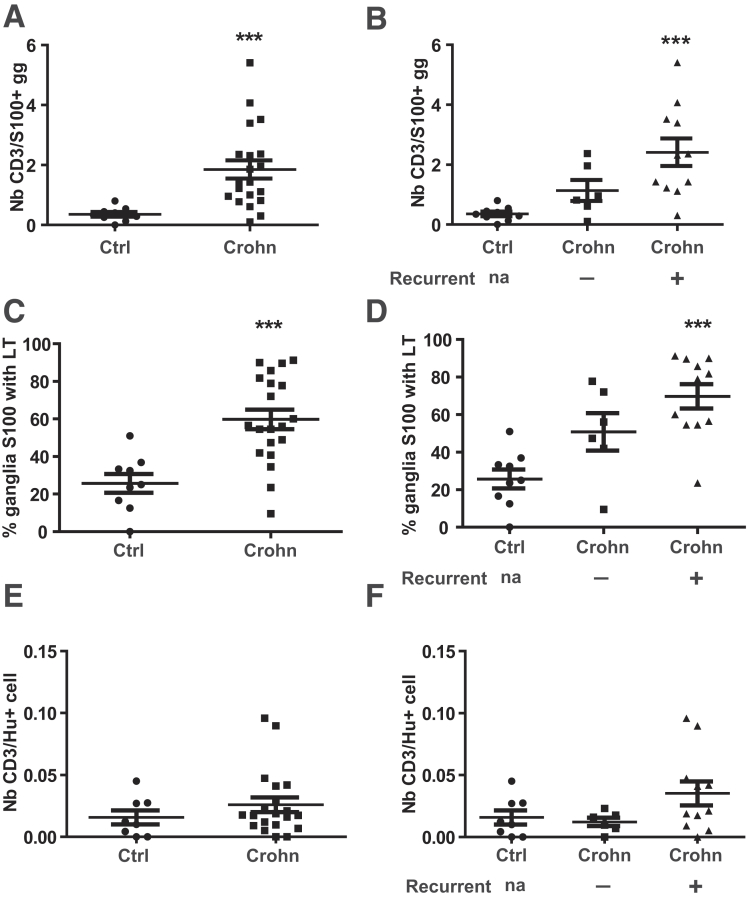
**T cells in myenteric ganglia of intestinal resections from control or Crohn’s disease patients.** Number of T cells was determined in control group of control (n = 9) or Crohn's disease patients (n = 20) **(***A, C, E***)**, but also in the control group of control (9), non-recurrent Crohn’s patients (n = 6), or recurrent Crohn's patients (n = 11) **(***B, D, F*). (*A* and *B*) Mean number of CD3+ T cells apposed to S100β staining per myenteric ganglia. (*C* and *D*) Percentage of myenteric ganglia with CD3+ T cells apposed to S100β staining. (*E* and *F*) Mean numbers of T cells apposed to Hu staining per Hu-positive cells. Data are mean ± standard error of the mean. Statistical analyses: Kruskall-Wallis followed by Dunn post hoc tests (*B, D, F*) or Mann-Whitney test (*A, C, E*). ∗∗∗*P* < .001.

We previously observed that proximal resection specimens from the gut of Crohn’s patients contain a higher number of T cells close to the glia in the myenteric ganglia as compared with control patients with cancer.^11^ To confirm this observation, serial sections prepared from the proximal margin resections of 9 control and 20 Crohn’s patients were double stained with anti-CD3 (T cells) combined with anti-S100β (glial cells) or anti-Hu (neurons) antibodies. Except for one control patient, CD3+ T cells were found close to S100β+ glia in all patients, and quantification studies confirmed a higher number of T cells apposed to glia in the myenteric ganglia of Crohn’s patients as compared with control subjects (Figure 1*A*). To evaluate a potential association between these observations and recurrence of the disease, the group of Crohn’s patients was split according to clinical and/or endoscopic recurrence at 18 months after surgical resection. The number of CD3+ cells apposed to myenteric glia was significantly higher in relapsing patients (2.42 ± 0.5) as compared with control patients (0.36 ± 0.08, *P* = .0007), whereas no significant difference was observed between controls and the group of patients still in remission (Figure 1*B*). The mean percentage of S100β+ ganglia with CD3+ cells was significantly higher in Crohn’s patients (58%, Figure 1*C*), especially in the group of patients with recurrence (70%, Figure 1*D*) as compared with control subjects (26%). We next aimed to determine whether T cells were apposed to the cell bodies of enteric neurons. Immunohistochemistry analyses revealed that the number of CD3+ cells apposed to HuD+ staining in control patients was similar to that found in the group of Crohn’s disease patients, whatever the postoperative recurrence status. No difference between these groups was detected (Figure 1*E* and *F*).

**Figure 2:**
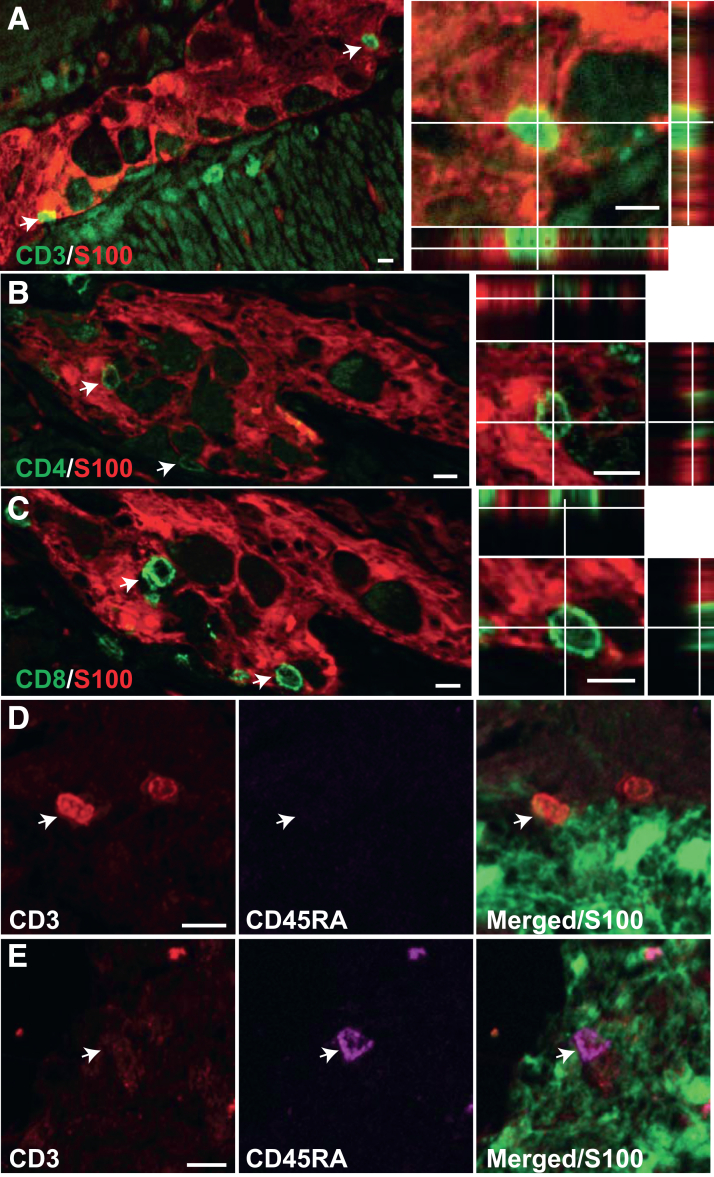
**Characterization of T cells apposed to glia in human myenteric ganglia.** (*A–C*) Representative apotome images with orthogonal reconstruction (*right panel*) showing CD3 (*A*), CD4 (*B*), and CD8 (*C*) cells apposed to glia. (*D* and *E*) Representative apotome images of CD3+CD45RA- (*D*) and CD3-CD45RA+ (*E*) cells apposed to S100 glia. *Arrows* point the cells of interest. In *D* or *E*, the *white arrows* point to the same cell in the three photomicrographs. Scale bar, 10 μm.

To characterize T cells in plexitis (Figure 2*A*), double immunostaining was performed, revealing the presence of both CD4 and CD8 apposed to S100β glia (Figure 2*B* and *C*) in all conditions. Triple staining with CD3, CD45RA, and S100β antibodies was also conducted to determine whether the T cells were naive, according to the group of patients (n = 3 per group). Analyses revealed that the CD3^+^ cells in or around the myenteric ganglia were all CD45RA^-^ (Figure 2*D*), indicating that T cells in plexitis were not naive, regardless of patient type**.** The rare CD3^-^CD45RA^+^ cells observed in plexitis were most probably B cells (Figure 2*E*).

### T-Cell Activation or Inflammatory Stimuli Increase T-Cell Adhesion to Glia

**Figure 3:**
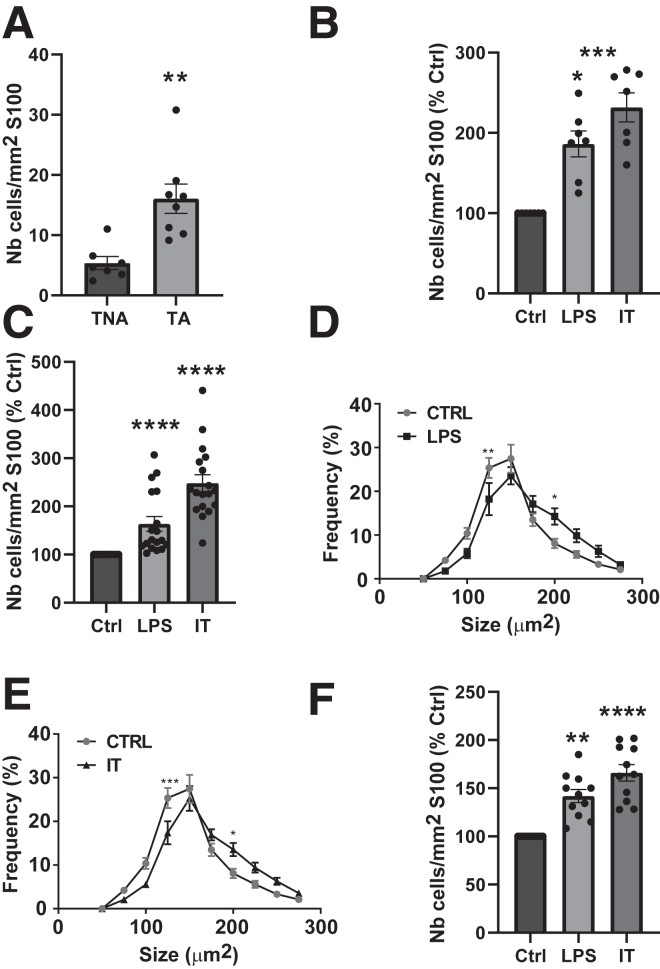
**Splenic and mesenteric T cells interact with myenteric glia in co-culture.** (*A*) Number of T cells adhering to glia after 2 hours of co-culture with non-activated (TNA, n = 7) or activated T cells (TA, n = 8). (*B* and *C*) Number of non-activated (*B*, n = 7) or activated (*C*, n = 18) CFSE+ T cells adhering to glia pretreated with LPS, IL-1β/TNFα (IT), or untreated (Ctrl). Number of adherent T cells is expressed relative to S100β surface and as percentage of control condition (% Ctrl). (*D* and *E*) Size frequency of activated T cells adhering to glia pretreated by LPS (*D*, n = 5) or IT (*E*, n = 5). (*F*) Number of activated mesenteric T cells adhering to glia untreated (Ctrl) or pretreated with LPS or IL-1β/TNFα (IT) (n = 11). Data are shown as mean ± standard error of the mean. Statistical analyses: Mann-Whitney test (*A*) or Kruskall-Wallis followed by Dunn post hoc tests (*B, C, F*) or 2-way analysis of variance (*D, E*). ∗*P* < .05, ∗∗P < .01, ∗∗∗*P* < .001, ∗∗∗∗*P* < .0001.

**Figure 4:**
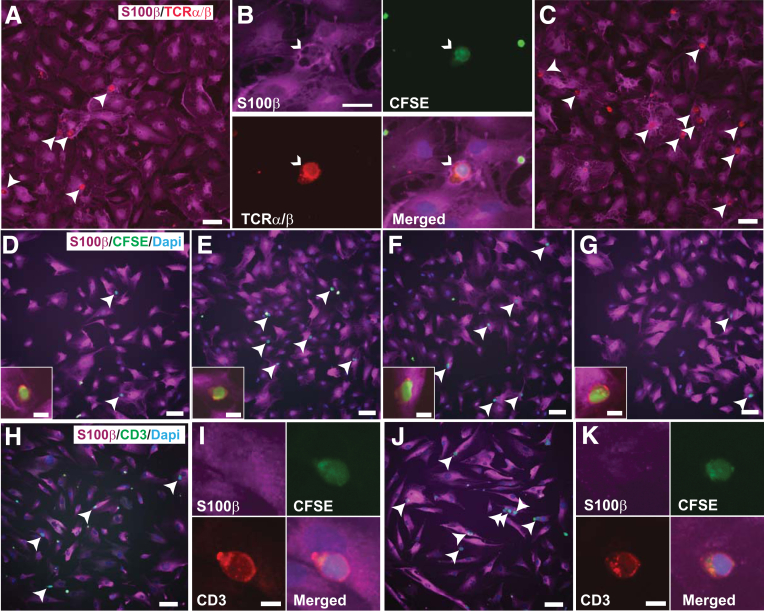
**Representative illustration of T-cell adhesion to enteric glia in co-cultures.** (*A–C*) Adhesion of activated splenic rat CD3 T cells to rat glia not pretreated (*A* and *B*) or pretreated with IT (*C*). (*D–G*) Adhesion of activated rat mesenteric CD3 T cells to rat glia not pretreated (*D*) or pretreated with IT alone (*E*), with solvent (Solv; *F*), or with LG (LG, *G*). (*H–K***)** Adhesion of activated human CD3 T cells from PBMC to human glia not pretreated (*H* and *I*) or pretreated with IT (*J* and *K*). All the photomicrographs were acquired after 2 hours of co-cultures. Staining, S100β, *purple* (*A–K*); CFSE, *green* (*A–G, I, K*); DAPI, *blue*; TCRα/β (rat), *green* (*B*, *D–G inset*), or CD3 (human), *green* (*H* and *J*) or *red* (*I* and *K*). *Arrowheads* point to T cells. In *B*, the *white arrowheads* point to the same spot in the four photomicrographs. Scale bar, *A* and *B*, 30 μm; *D* and *E*, 60 μm; insert, 20 μm; *H* and *J*, 60 μm; *I* and *K*, 60 μm.

To determine whether T-cell activation could modulate the interactions between T cells and glia, purified rat glia were co-cultured for 2 hours with splenic T cells, non-activated or activated for 3 days with CD3/CD28 antibodies. The number of T cells adhering to glia was then counted and expressed relative to the S100β surface. A greater number of adherent T cells was found when the lymphocytes were previously activated (Figure 3*A*). Similarly, to evaluate the impact of proinflammatory molecules on glia–T-cell interactions, glia were pretreated or not for 24 hours with lipopolysaccharides (LPS) or IL-1β/tumor necrosis factor alpha (TNFα). The supernatants were then removed, and non-activated or activated T cells were added to glia. After 2 hours of co-cultures, we observed a higher number of T cells adhering to pretreated glia (Figure 4*A–C*), whether the T cells were activated or not (Figure 3*B* and *C*). T-cell spreading, considered as a marker of their activation, was also enhanced because an increase in the size of activated T cells was observed in the presence of glia pre-exposed to LPS or IL-1β/TNFα (Figure 3*D* and *E*). Importantly, similar results were obtained with T cells isolated from the mesenteric lymph nodes (Figures 3*F* and 4*D* and *E*).

### ICAM-1/LFA-1 Are Implicated in the Adhesion of T Cells to Rat Glia

**Figure 5:**
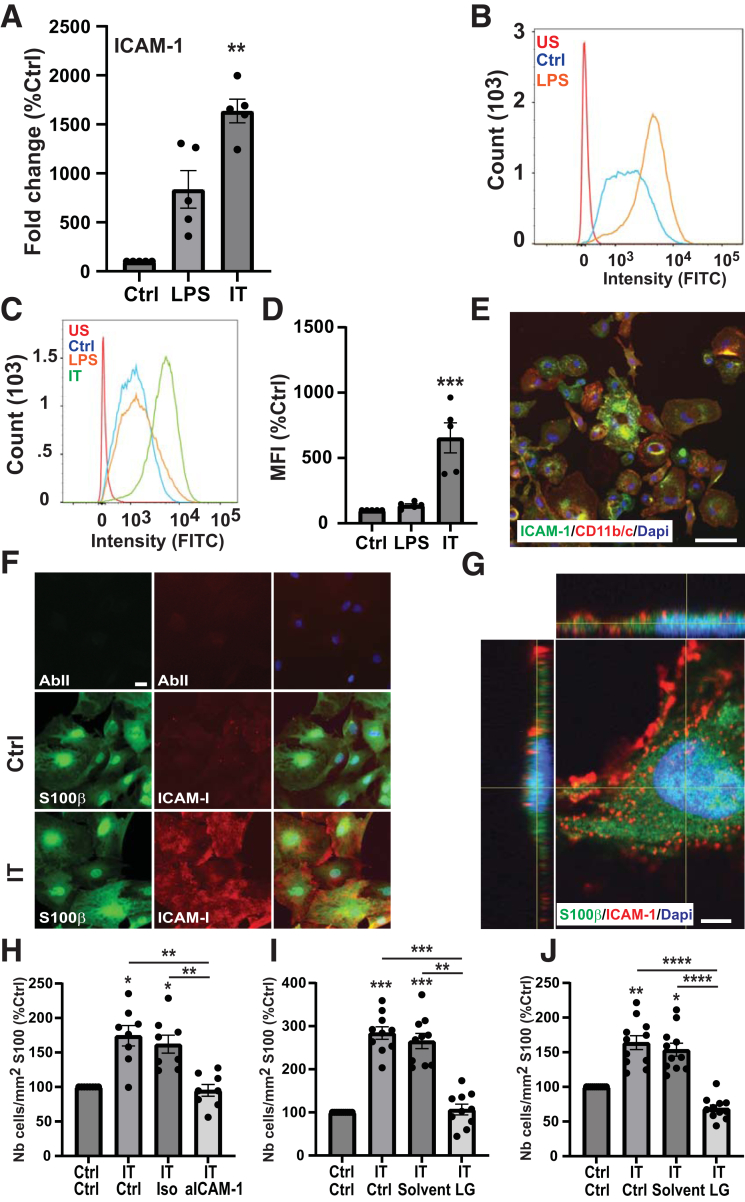
**ICAM-1 is expressed in rat myenteric glia and is involved in glia–T-cell adhesion.** (*A*) Quantitative polymerase chain reaction analyses of ICAM-1 mRNA in glia untreated (Ctrl) or treated for 6 hours with LPS or IT (n = 5 per group). (*B* and *C*) Analysis of ICAM-1 expression by flow cytometry. Rat macrophages used as positive control (*B*) and rat enteric glia (*C*) were treated or not for 24 hours with proinflammatory stimuli and then immunolabeled with anti–ICAM-1 antibody. The intensity of fluorescence (FITC) was measured by flow cytometry. US, unstained cells. (*D*) Relative abundance of ICAM-1 in treated or untreated glia was then determined by calculating the mean intensity of fluorescence (MIF) for each condition (n = 5 per group). (*E*) Photomicrograph of macrophages treated for 24 hours with LPS, immunostained with CD11b/c (*red*) and ICAM-1 (*green*) antibodies, and counterstained with DAPI (*blue*). Scale bar, 60 μm. (*F*) Micrograph of glia treated for 24 hours with IT (IT) or not (Ctrl) and then immunostained or not (AbII) with anti-S100β (*green*) and anti–ICAM-1 (*red*) antibodies. Nuclei were counterstained with DAPI (*blue*). Scale bar, 20 μm. (*G*) Confocal micrograph with Z reconstruction of glia treated with IT for 24 hours and immunostained with S100β (*green*) and ICAM-1 (*red*) antibodies. Nuclei were counterstained with DAPI (*blue*). Scale bar, 5 μm. (*H–J*) Effect of LFA-1/ICAM-1 neutralization on glia–T-cell interactions. Glia pretreated with IT were preincubated for 60 minutes with anti–ICAM-1 antibody or isotype control (Iso) and co-cultured for 2 hours with splenic activated T cells (*H***,** n = 8). Activated T cells from spleen (*I*, n = 10) or mesenteric lymph nodes (*J*, n = 11) were preincubated with solvent (Solv) or LG and co-cultured for 2 hours with LG-treated glia. The number of CFSE+T cells adhering to glia was expressed relative to S100β surface and as percentage of control condition. Data are shown as mean ± standard error of the mean. Statistical analyses: Kruskall-Wallis followed by Dunn post hoc tests. ∗*P* < .05, ∗∗*P* < .01, ∗∗∗*P* < .001, ∗∗∗∗*P* < .0001.

With the aim of identifying molecular mechanisms involved in the T-cell adhesion to glia, we investigated the regulation of ICAM-1 previously identified as involved in T-cell interaction with other cell types. Quantitative polymerase chain reaction and flow cytometry analyses indicated that treatment of glia for 6 or 24 hours with IL-1β/TNFα up-regulated ICAM-1 mRNA (Figure 5*A*) and protein (Figure 5*C* and *D*), respectively. This observation was confirmed by immunocytochemistry because a weak ICAM-1 immunoreactivity was observed in control condition, whereas a strong expression of the adhesion molecule was found in glia treated for 24 hours with IL-1β/TNFα (Figure 5*F*). Imaging by confocal microscopy revealed a punctiform non-homogeneous expression of ICAM-1 in the cell membrane of S100β-positive cells treated with IL-1β/TNFα (Figure 5*G*).

To evaluate the potential involvement of ICAM-1 in the T-cell adhesion to glia, ICAM-1/LFA-1 binding was blocked with an anti–ICAM-1 neutralizing antibody or with lifitegrast (LG), a chemical inhibitor of LFA-1. As presented in Figure 5*H*, treatment of glia with anti–ICAM-1 antibody decreased the number of activated splenic T cells adhering to glia pretreated with IL-1β/TNFα. A decrease was also observed after the treatment of glia and activated T cells with LG (Figure 5*I*). Importantly, this result was confirmed with T cells derived from the mesenteric lymph nodes (Figures 4*D–G* and 5*J*).

### ICAM-1/LFA-1 Are Implicated in T-Cell Adhesion to Human Glia

**Figure 6:**
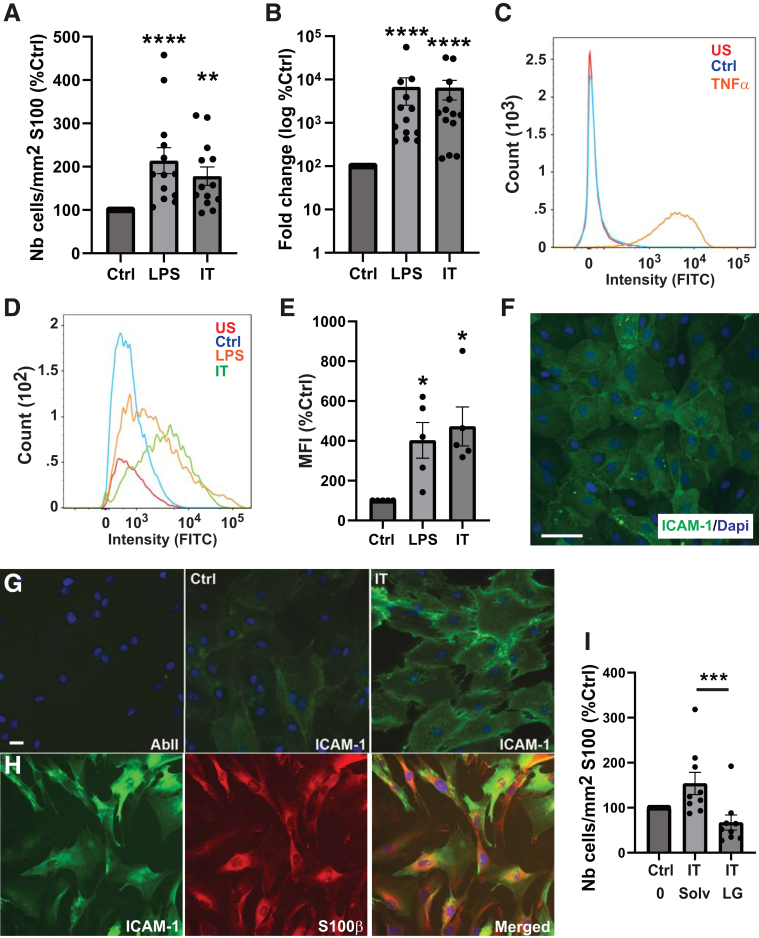
**Activated T cells interact with human glia in vitro: involvement of ICAM-1.** (*A*) Number of human activated T cells adhering to glia after 2 hours of co-culture with human glia pretreated for 24 hours with LPS (LPS), IT, or untreated (Ctrl) (n = 11). (*B*) Expression of ICAM-1 in human glia analyzed by quantitative polymerase chain reaction (n = 11). (*C* and *D*) Flow cytometry analysis of ICAM-1 expression in human dermal microvascular endothelial cells (*C*, HDMEC, positive control cells) treated or not by TNFα for 24 hours and in glia (*D*) treated or not by LPS or IT for 24 hours. US, unstained cells. (*E*) Relative abundance of ICAM-1 in treated or untreated glia was determined by calculating the MIF for each condition (n = 5 per group). (*F*) Photomicrograph of HDMEC (ICAM-1 positive control cells) treated for 24 hours with TNFα, stained with ICAM-1 antibody (*green*) and counterstained with DAPI (*blue*). Scale bar, 60 μm. (*G*) Photomicrograph of glia treated for 24 hours with IT or not (Ctrl) and then immunostained or not (AbII) with anti–ICAM-1 (*green*) antibody. (*H*) Photomicrograph of glia treated for 24 hours with IT and then double stained with anti-S100β (*red*) and anti–ICAM-1 (*green*) antibodies. Nuclei were counterstained with DAPI (*blue*). Scale bar, 10 μm. (*I*) Effect of LFA-1/ICAM-1 neutralization on glia–T-cell interactions. Activated T cells were preincubated for 30 minutes with solvent (Solv) or LG before their addition to glia pretreated (IT) or not (Ctrl) for 24 hours with IT. Glial cells were pretreated with solvent or LG at the same time and in the same manner as T cells. The number of T cells adhering to glia was counted after 2 hours of co-culture (n = 9). Results are expressed relative to S100β surface and as percentage of control condition. Data are shown as mean ± standard error of the mean. Statistical analyses: Kruskall-Wallis followed by Dunn post hoc tests. ∗∗*P* < .01, ∗∗∗*P* < .001, ∗∗∗∗*P* < .0001.

Because cell interactions and molecular pathways may differ between humans and rodents, in vitro investigations were carried out with human glia and blood-derived T cells activated for 6 days with anti-CD3 and anti-CD28 antibodies. We showed that pretreatment of human glia with LPS or IL-1β/TNFα increased the number of adhering T cells in co-cultures (Figures 4*H–J* and 6*A*) and up-regulated the expression of ICAM-1 mRNA (Figure 6*B*) and protein (Figure 6*D* and *E*) in glia. Immunocytochemistry confirmed that ICAM-1 is expressed by human glia in basal conditions and is strongly up-regulated after 24 hours of treatment with IL-1β/TNFα (Figure 6*G* and *H*). Interestingly, as observed in rat glia, the pretreatment of human glia and T cells with the LFA-1 chemical inhibitor decreased the number of T cells adhering to glia in co-cultures (Figure 6*I*).

To determine whether ICAM-1 could be detected in glia from human biopsies, single-cell data sets from healthy and Crohn’s patients were analyzed.^13^ As shown in Table 1, ICAM-1 was detected in glial cells from human biopsies, and a higher number of ICAM-1–expressing glia was observed in inflamed samples from Crohn’s patients. However, using pseudo-bulk generation, no statistically significant increase in ICAM-1 expression levels was found in inflamed or non-inflamed samples from Crohn’s disease patients as compared with healthy individuals in both colon and terminal ileum.

**Table 1:** Analysis of ICAM-1 Expression in Glia From Human Colon or Terminal Ileum Using Single-Cell RNA Sequencing data^13^

	Colon	Terminal ileum
Healthy glia	1211	502
Healthy glia expressing ICAM-1 (%)	2.8	1.2
Non-inflamed glia	934	1636
Non-inflamed glia expressing ICAM-1 (%)	2.9	4.0
Log2FC (healthy vs von-inflamed)	0.176697	–0.619624
*P* value	.82979	.485643
*P* value adjusted	.898018	NA^a^
Inflamed cells	72	479
Inflamed glia expressing ICAM-1 (%)	13.9	5.6
Log2FC (healthy vs inflamed)	1.01513	0.480996
*P* value	.374382	.650252
*P* value adjusted	.62963	NA^a^

a Row has a low mean normalized count.

**Figure 7:**
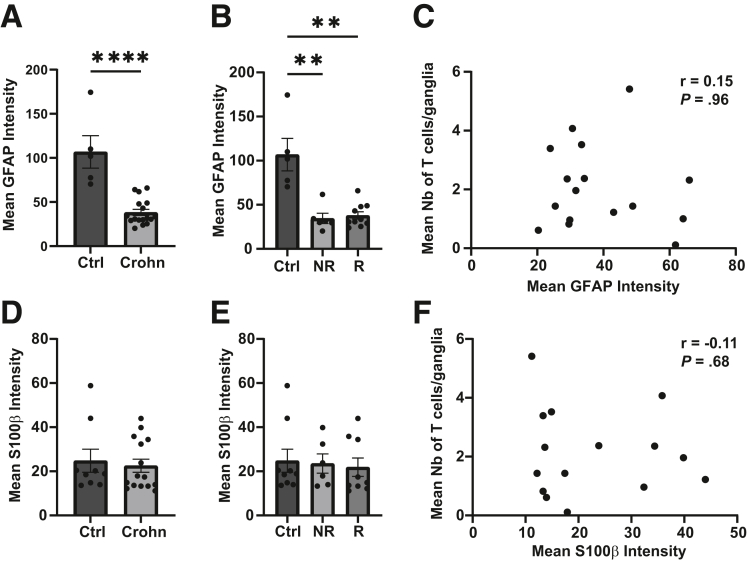
**Correlation between T-cell plexitis and ganglia glial marker intensity.** (*A* and *B*) Intensity of GFAP staining in the myenteric ganglia of control (Ctrl), Crohn, recurrent (R), and non-recurrent (NR) patients. (*C*) Correlation between number of T cells in the myenteric ganglia and the mean GFAP intensity in the ganglia. (*D* and *E*) Intensity of S100β staining in the myenteric ganglia of control (Ctrl), Crohn, recurrent (R), and non-recurrent (NR) patients. (*F*) Correlation between number of T cells in myenteric ganglia and mean S100β intensity of the ganglia. (*A*, *B*, *D*, and *E*) Data are shown as mean ± standard error of the mean. Statistical analyses: Kruskall-Wallis followed by Dunn post hoc tests. ∗∗*P* < .01, ∗∗∗∗*P* < .0001. (*C* and *F*) Statistical analyses: Spearman analysis. r, correlation coefficient.

We next checked the possibility of a correlation between glia activation and an increased number of T cells in the myenteric plexus from Crohn’s patients. In this aim, intensity of the GFAP and S100β^14^ staining in ganglia from the proximal resection margin was analyzed, because these 2 glial markers were reported to be increased in case of inflammation.^14^ A decrease in the intensity of GFAP staining was observed in the ganglia from Crohn’s patients as compared with controls, but no difference was found between recurrent and non-recurrent Crohn’s patients (Figure 7*A* and *B*). Further analyses did not reveal a potential correlation between the mean number of T cells per ganglion and the intensity of GFAP staining in Crohn’s patients (Figure 7*C*). Concerning S100β, no difference in the intensity could be detected between the different groups (Figure 7*D* and *E*). In addition, no correlation between S100β intensity and the mean number of T cells per ganglion in Crohn’s patients was observed (Figure 7*F*).

### Treatment With LG Decreases Colitis in 12-Week-Old Winnie Mice

**Figure 8:**
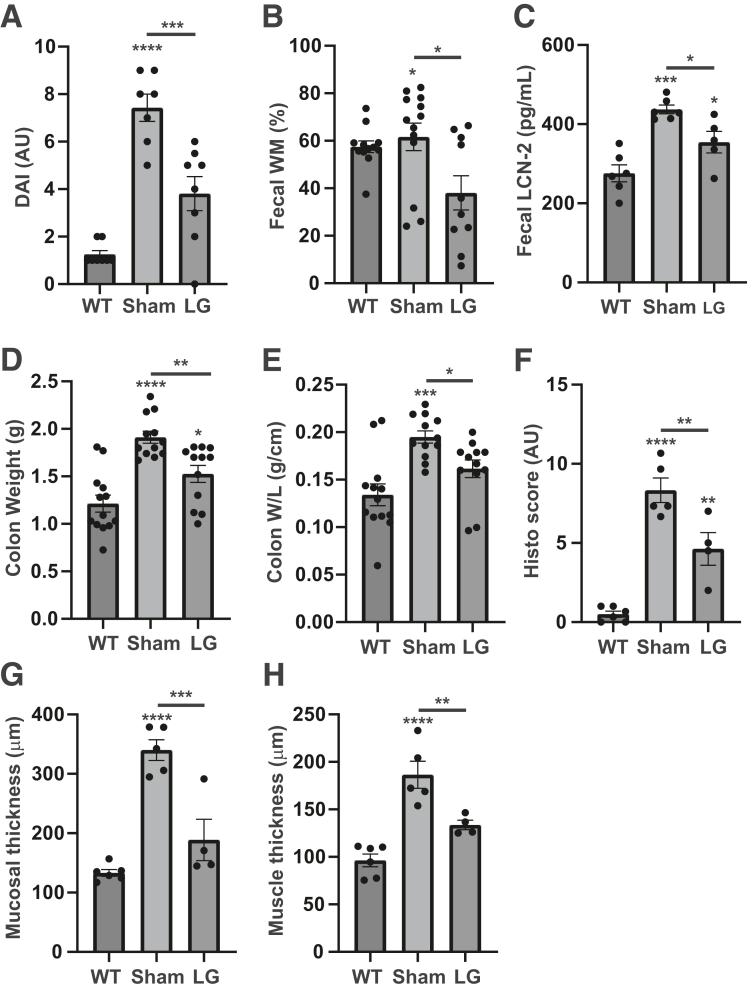
**Effect of LG on colitis and distal colonic motility in Winnie mice.** Twelve-week-old Winnie mice with active colitis received intraperitoneal injection of solvent (Sham) or 15 mg/kg LG twice a day for 14 days. Then colitis and distal colonic motility were compared with the data obtained in aged-matched WT mice. (*A*) Disease activity index (WT, n = 8; Sham, n = 7; LG, n = 8). (*B*) Day14 fecal wet mass (WT, n = 12; Sham, n =13; LG, n = 10). (*C*) Day 14 fecal Lcn-2 concentration (WT, n = 6; Sham, n = 6; LG, n = 5). (*D*) Colon weight (WT, n = 13; Sham, n = 12; LG, n = 12). (*E*) Colon weight/length ratio (WT, n = 13; Sham, n = 12; LG, n = 12). (*F–H*) Histologic analyses of the distal colon: global scoring (*F*), mucosal (*G*) and muscle (*H*) thickness (Ctrl, n = 6; Sham, n = 5; LG, n = 4). Data are shown as mean ± standard error of the mean. Statistical analyses: one-way analysis of variance followed by Tukey’s multiple comparisons tests. ∗*P* < .05, ∗∗*P* < .01, ∗∗∗*P* < .001, ∗∗∗∗*P* < .0001.

**Figure 9:**
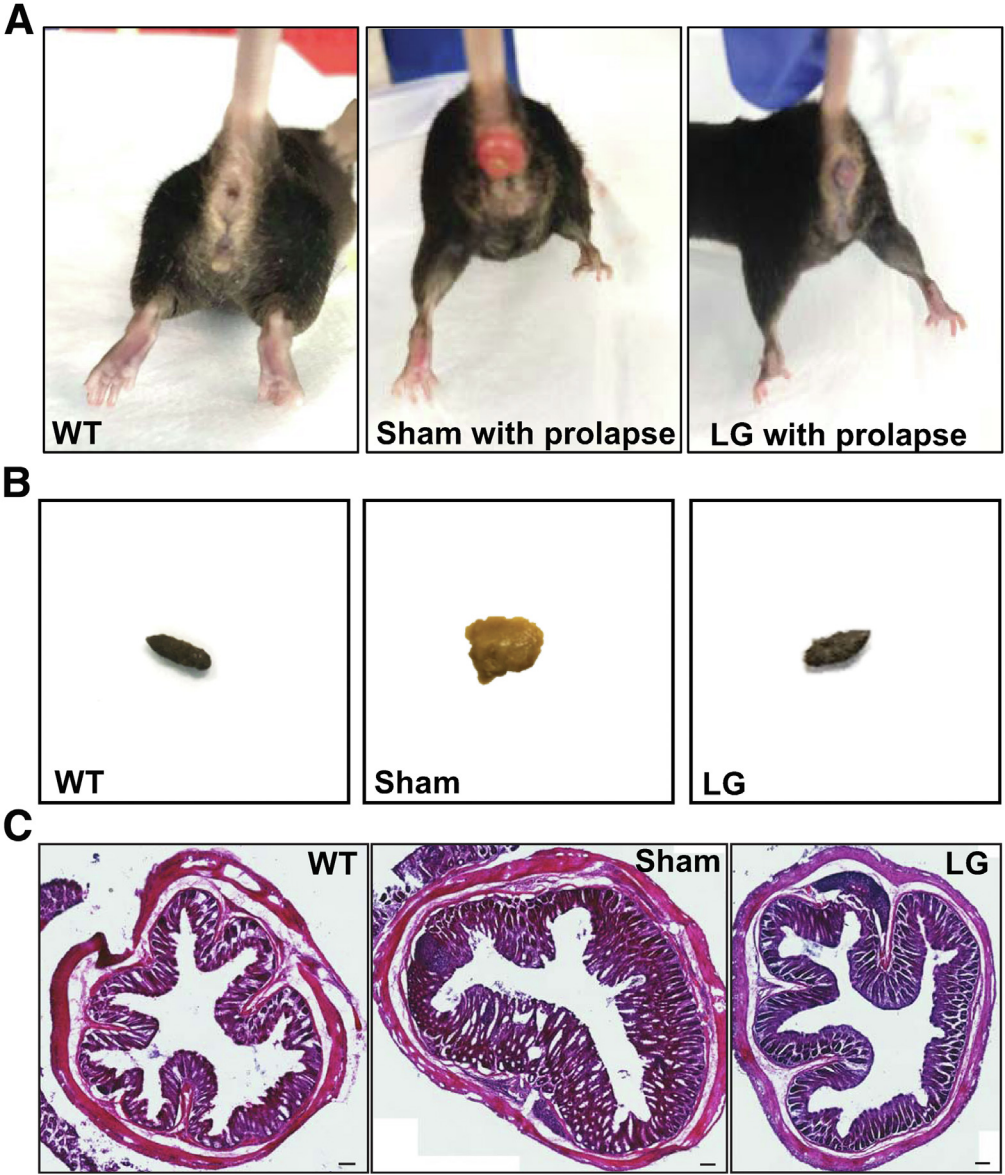
**Clinical manifestations and histologic observations.** (*A*) Representative images of WT as well as sham- and LG-treated Winnie mice with rectal prolapse. (*B*) Representative images of fecal pellets. (*C*) Representative hematoxylin-eosin staining of distal colon cross sections.

To evaluate the in vivo impact of LFA-1/ICAM-1 neutralization on active colitis and on the formation of myenteric plexitis, LFA-1 chemical inhibitor was administered to Winnie mice that spontaneously develop colitis and plexitis at the age of 2 months.^15^ For this purpose, 12-week-old Winnie mice were injected intraperitoneally with 15 mg/kg LG twice a day for 14 days. Such treatment led to 51% decrease in the disease activity index as compared with the sham-treated group (Figure 8*A*). We also observed a significant diminution of several parameters including the fecal water content (Figure 8*B*), the concentration of fecal lipocalin-2 (Figure 8*C*), the distal colon weight (Figure 8*D*), the weight/length ratio (Figure 6*E*), the histologic score (Figure 8*F*), as well as the thickness of the mucosa and muscle layers (Figure 6*G* and *H*). Representative illustrations of the clinical and histologic changes are available in Figure 9*A–C*.

### Treatment With LG Partially Restores the Frequency of Colonic Migrating Motor Complexes in 12-Week-Old Winnie Mice

**Figure 10:**
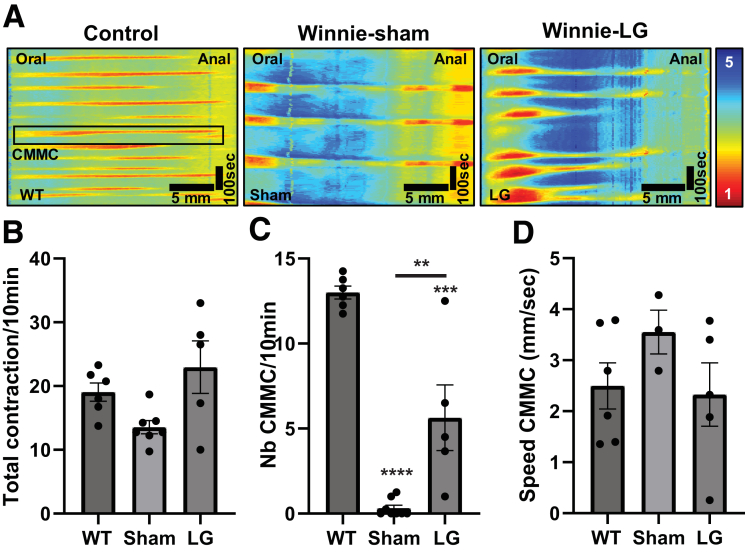
**Effect of LG on distal colonic motility in Winnie mice**. Twelve-week-old Winnie mice with active colitis received intraperitoneal injection of solvent (Sham, n = 8) or 15 mg/kg LG (n = 5) twice a day for 14 days. Distal colonic motility was then compared with the data obtained in aged-matched WT mice (n = 6). (*A*) Representative spatiotemporal maps of colons from WT and sham- or LG-treated Winnie mice. (*B*) Frequency of total contractions. (*C* and *D*) Frequency (*C*) and speed (*D*) of CMMC. Data are shown as mean ± standard error of the mean. Statistical analyses: one-way analysis of variance followed by Tukey’s multiple comparisons test. ∗*P* < .05, ∗∗*P* < .01, ∗∗∗*P* < .001, ∗∗∗∗*P* < .0001.

To evaluate the impact of LG on colonic motility, the whole distal colons of sham- and LG-treated Winnie mice were collected and analyzed ex vivo. Video imaging and spatiotemporal mapping were performed to quantify the frequency and speed of the contractions (Figure 10*A*). The analyses revealed a strong decrease in the frequency of the colonic migrating motor complexes (CMMC) in sham-treated Winnie mice. Indeed, a mean of 0.31 ± 0.18 CMMC/10 min was reported with distal colons from sham-treated Winnie mice, whereas a mean of 13 ± 0.38 CMMC/10 min was recorded with distal colons from wild-type (WT) mice (Figure 10*C*). Importantly, the administration of LFA-1 antagonist partially reversed this low frequency, with a mean of 5.63 ± 1.93 CMMC/10 min observed in the Winnie mice treated with LG twice a day for 14 days (Figure 10*C*). Concerning the speed of propagation of the detected CMMC, no statistical difference was observed between the 3 groups (Figure 10*D*).

### Treatment With LG Decreases Plexitis in 12-Week-Old Winnie Mice

**Figure 11:**
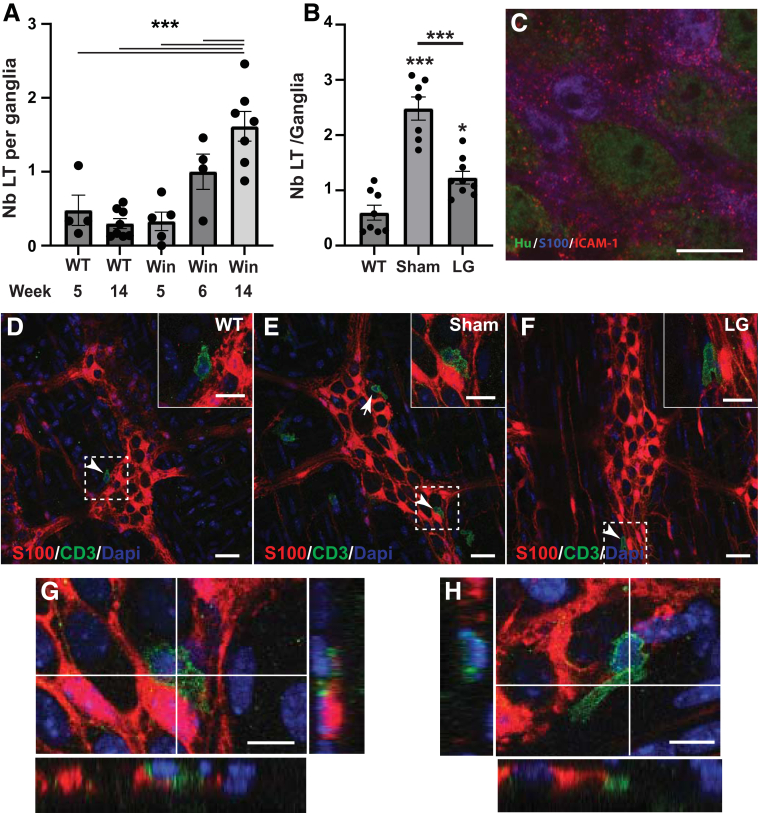
**Effect of LG on T-cell infiltration in myenteric plexus of Winnie mice.** (*A*) Number of T lymphocytes in the distal colon myenteric ganglia of WT mice at 5 (n = 4) and 14 (n = 8) weeks of age and of Winnie mice (Win) at 5 (no symptoms, n = 5), 6 (first colitis, n = 4), and 14 (active colitis, n = 7) weeks of age. (*B*) Effect of LG in Winnie mice. Twelve-week-old Winnie mice with active colitis received solvent (Sham) or 15 mg/kg LG twice daily for 14 days. Immunohistochemistry was performed on LMMP preparations (WT, n = 8; Sham, n = 7; LG, n = 9). Number of T lymphocytes (CD3+ DAPI+) in contact with S100+ cells was then counted per ganglia. (*C*) Expression of ICAM-1 in myenteric ganglia of Winnie mice. Immunohistochemistry with anti–ICAM-1 antibody shows a punctiform expression of ICAM-1 (*red*) co-localized with S100β^+^ enteric glial cells (*purple*) and Hu^+^ neurons (*green*). Scale bar, 10 μm. (*D–F*) Representative confocal micrographs showing CD3+ lymphocytes (*green*) in myenteric plexus of WT mice and of Sham- and LG-treated Winnie mice, as determined with anti-CD3 (*green*) and anti-S100β (*red*) antibodies. Nuclei were counterstained with DAPI (*blue*). Scale bar, 30 μm. (*G* and *H*) Z reconstruction of confocal images from *E* showing T cells (*green*) apposed to glia (*red*). Arrows point to T cells apposed to myenteric ganglia. Scale bar, 10 μm. Data are shown as mean ± standard error of the mean. Statistical analyses: one-way analysis of variance followed by Tukey’s multiple comparisons test. ∗*P* < .05, ∗∗*P* < .01, ∗∗∗*P* < .001, ∗∗∗∗*P* < .0001.

We next determined the impact of LFA-1 antagonist on plexitis by analyzing T-cell infiltration in the distal colon of sham- or LG-treated Winnie mice. First, T-cell infiltration was evaluated in the myenteric plexus of Winnie mice aged 5 (no symptoms), 6 (start of colitis), or 14 (active colitis) weeks to control the apparition of plexitis. For this purpose, S100β and CD3 immunohistochemistry was performed on longitudinal muscle-myenteric plexus (LMMP) preparations, and the number of intraganglionic T lymphocytes apposed to S100β^+^ cells was determined (Figure 11*A*). The results indicated a higher number of T cells close to S100β^+^ cells in 14-week-old Winnie as compared with WT mice or asymptomatic 5-week-old Winnie. A 14-day treatment of 12-week-old Winnie mice with LG induced a 2-fold decrease in the number of T cells close to glia as compared with untreated Winnie mice (Figure 11*B*, *D–H*). The expression of ICAM-1 was controlled by double immunohistochemistry using S100β and ICAM-1 antibodies. As shown in Figure 11*C*, punctiform expression of ICAM-1 was observed in S100β+ glia in the myenteric plexus of Winnie mice.

## Discussion

Comparative analyses of the myenteric plexus at the proximal ileal resection margins show that Crohn’s patients with postoperative recurrence present a higher number of T cells close to glia as compared with control patients. Interestingly, in vitro investigations reveal that T-cell activation and/or inflammatory stimuli favor the adhesion of T cells to human or rat glia. The adhesion molecule ICAM-1 is probably implicated in these interactions as supported by its increased expression in glia in response to proinflammatory stimuli and the decreased number of adherent T cells after the blockage of ICAM-1/LFA-1 interactions. This view is reinforced by the fact that systemic inhibition of LFA-1 with LG reduces myenteric plexitis and colitis in a mouse model of spontaneous colitis.

Plexitis at the proximal margin of intestinal resections is recognized as a predictive marker of early postoperative recurrence. However, its current use is limited because of the variety of immune cells listed in the literature but also the difficulty of identifying plexitis with basic hematoxylin-eosin staining. In the present study, we therefore restricted our investigation to T-cell infiltrates and used double immunohistochemistry to only count T cells close to neural cells. We found T cells in the myenteric ganglia of patients with cancer or Crohn’s disease as previously reported.^16^^,^^17^^,^^18^ In addition, we clearly showed that these infiltrating T cells were apposed to Hu-positive neurons and S100β-positive glia. Interestingly, a higher number of T cells close to glia was observed in the myenteric ganglia from Crohn’s patients (all considered together and those with postoperative recurrence) as compared with patients with cancer. These results confirm our preliminary observations.^11^ They also point out that neuroimmune interactions can occur in the myenteric plexus of steady-state patients and then increase in case of inflammation. Studies with a higher number of patients are required to determine the cutoff value beyond which patients have a high risk of recurrence and then should follow a preventive treatment. In this aim, double immunohistochemistry against markers of immune and neural cells would be of a great help to quantify plexitis and standardize their evaluation.

Our present in vitro studies revealed that T-cell adhesion to glia increased if the glial cells were previously exposed to LPS or IL-1β/TNFα. Changes in the phenotype of glia in reaction to proinflammatory stimuli were previously observed,^19^ and our present data suggest that such plasticity may favor the formation of plexitis in case of Crohn’s disease. Indeed, tight-junction and epithelial alterations in the intestinal barrier in case of mild active Crohn’s disease^20^ have been suggested to favor the entrance of bacterial fragments in addition to other ways of the bacterial invasion.^21^ Glia may then be exposed to bacterial fragments and LPS, which may in turn favor T-cell adhesion to glia. Moreover, because glia can produce IL-1β and TNFα in response to LPS,^22^ an amplification loop may occur, increasing glia–T-cell interaction in the myenteric plexus. Interestingly, in situ hybridization on intestinal mucosal biopsies from IBD patients revealed increased expression of IL-1β and TNFα in epithelial and lamina propria cells in active zones but also in non-active regions.^23^ This local production of proinflammatory cytokines may favor the accumulation of T cells at the proximity of glia in inflamed regions but also in non-active zones. In the present work, we were unable to correlate the mean intensity of S100 or GFAP staining in the myenteric ganglia to the mean number of T cells apposed to glia, but the possibility of a heterogeneous distribution of inflammation within the proximal resection margin must be considered when interpreting such finding. In this perspective, a combined analysis of the two parameters on ganglion-by-ganglion basis using whole-mount preparations could be highly informative.

One possibility concerning the changes that favor glia–T-cell interaction is an increased expression of adhesion molecules in glia. Indeed, we provide the first evidence that exposure of rat or human glia to IL-1β and TNFα stimulates the production of ICAM-1. Similar up-regulation was previously observed in cultures of human astrocytes.^24^ Induction of ICAM-1 in glia in response to inflammatory stimuli remains to be determined in vivo, but increased production of ICAM-1 was reported in case of Crohn’s disease,^25^ and an up-regulation of ICAM-1 mRNA was observed in the whole enteric nervous ganglia of Crohn’s patients refractory to treatment.^26^ Using single-cell RNA sequencing data set,^13^ we were able to detect ICAM-1 in a subset of glia, but no statistically significant difference in its expression could be detected in colon or terminal ileum samples from Crohn’s patients compared with healthy individuals. However, this observation must be interpreted with caution because single-cell RNA sequencing raw counts do not provide an absolute measure of gene expression within a given sample. Indeed, genes with low expression level such as ICAM-1 are less likely to be detected and often need much higher sequencing depth.

To examine a potential implication of ICAM-1 in the adhesion of T cells to glia, blocking experiments were carried out. The decreased number of adherent T cells after the exposure of rat glia to anti–ICAM-1 antibodies supports an implication of this adhesion molecule in glia–T-cell interaction. Similar results were observed in cultures of astrocytes, except that both antibodies against ICAM-1 and vascular cell adhesion molecule-1 were necessary to reduce the percentage of T cells adhering to astrocytes.^27^

ICAM-1 plays a key role in firm adhesive interaction through its binding to LFA-1. This integrin, constitutively expressed by T cells in an inactive form, acquires high affinity for ICAM-1 upon T-cell receptor or chemokine receptor stimulation.^28^ Such changes most probably contribute to the increased number of T cells adhering to glia after T-cell activation with CD3/CD28 antibodies. An implication of ICAM-1/LFA-1 in glia–T-cell interaction is further supported by the fact that in vitro treatment of activated T cells with LG partially inhibits their adhesion to glia.

On the basis of these observations, LG was administered to Winnie mice, which spontaneously develop plexitis and colitis. The results revealed that the LFA-1 antagonist reduced the number of plexitis but also the severity of colitis. These data highlight the importance of LFA-1/ICAM-1 interactions in these two processes, even if it remains to be determined whether only glial ICAM-1 is implicated in the formation of plexitis and whether enteric plexitis are involved in the development of colitis. In this perspective, experiments using a loxed ICAM-1 animal crossed with inducible Cre under a glial promotor in a Winnie phenotype would be necessary in particular to assess the role of glial ICAM-1 in the formation of plexitis and its possible contribution to the development of colitis.

Concerning the restorative impact of LG on colonic motor function, the local T-cell depletion in the muscular layer and/or myenteric plexus may contribute to the improvement of intestinal motility in the treated Winnie mice. Further experiments are required to decipher the mechanism, but interferon-gamma–producing Th1 cells have been shown to inhibit motor function in postoperative ileus,^29^^,^^30^ and high amount of opioids known for their impact on motility have been observed in colonic T cells from colitis models motility.^31^^,^^32^

In previous attempts of ICAM-1/LFA-1 neutralization, some beneficial effects were observed in animal models, leading to clinical trials based on the administration of antisense oligonucleotides targeting ICAM-1 (Alicaforsen). After promising phase II clinical trials, larger clinical trials did not demonstrate a significant efficacy of this therapeutical approach possibly because of the low availability of oligonucleotides in targeted tissues but also the redundancy of the adhesion molecules involved in leukocytes recruitment.^33^ Thus, our strategy was to target LFA-1 with LG, an antagonist already approved in dry eye disease therapy.^34^ Systemic administration of LG in a curative way for 14 days in the Winnie mice shows beneficial effects such as reduction of inflammation, dysmotility, and colitis symptoms, but also a lower amount of T cells apposed to glia in the myenteric plexus.

In complement to the demonstration that more T cells are apposed to glia in case of Crohn’s disease, the functional consequences of this cell adhesion remain to be deciphered. Studies on glia of the central nervous system showed that the interactions between T cells and astrocytes contribute to increased ectonucleotidase in CD4+ T cells associated with an immunosuppressive-like phenotype^27^ and exert a cytotoxic action on astrocytes without induction of T-cell proliferation.^35^ Thus, deciphering the consequences of glia–T-cell interactions on their respective phenotype may help in understanding the mechanisms implicated in the development and recurrence of Crohn’s disease.

## Materials and Methods

All authors had access to the study data and had reviewed and approved the final manuscript.

Methods and data sets presented in the different figures of the article are available upon request.

### Human Tissues

Paraffin blocks of ileocolonic resections (proximal margin) from 24 patients operated in 2016 were collected, and 5-μm sections were cut using a microtome. All the procedures were performed according to the guidelines of the French Ethics Committee for Research on Humans (DC-2008-402). The sociodemographic, clinical, histologic, and endoscopic data of patients with Crohn’s disease are detailed in Table 2. Four patients were excluded because of the absence (n = 3) or low number of myenteric ganglia (<10, n = 1). Postoperative recurrence was assessed by colonoscopy (Rutgeerts score ≥ i1) within 1 year after surgery and/or clinical evaluation (physical examination) at 18 months after surgery. Three patients were lost for follow-up at 18 months (1 patient died 10 days after surgery, 2 patients did not have any colonoscopy). Nine patients who underwent ileocolonic resections for colorectal cancer in 2016 were included as controls (Ctrl). Sections were performed at the proximal margin away from the tumor tissue.

**Table 2:** Sociodemographic, Clinical, Histologic, and Endoscopic Data From Patients With Crohn’s Disease

Demographics, clinical, histologic, and endoscopic data	Total (n = 24)
Sex, n (%)	
Male	11 (45.8)
Age at diagnosis (y), median [IQR]^a^	24.0 [20.0–41.8]
Disease duration before first ileocolonic resection (y), median [IQR]^a^	2.0 [0.8–8.5]
Age at index resection (y), median [IQR]	42.0 [27.5–49.5]
Previous surgery, n (%)	6 (25.0)
Disease location, n (%)^b^	
Small bowel (L1)	10 (41.7)
Colonic (L2)	2 (8.3)
Ileocolonic (L3)	11 (45.8)
Upper digestive tract (L4)	0 (0.0)
Perianal disease (p), n (%)^b^	
Inactive lesions	2 (8.3)
Active lesions (Cardiff classification)	
Ulcerations	0 (0.0)
Fistula/Abscess	1 (4.2)
Stricture	1 (4.2)
Disease behavior, n (%)^b^	
Inflammatory (B1)	1 (4.2)
Stricturing (B2)	16 (66.7)
Penetrating (B3)	6 (25.0)
Smoking status at time of index surgery, n (%)^c^	
Never smoker	5 (20.8)
Past smoker	5 (20.8)
Active smoker	11 (45.8)
CRP >5 mg/L at time of index surgery, n (%)^d^	8 (41.7)
Surgical conditions	
Emergency surgery, n (%)	6 (25.0)
Elective surgery, n (%)	18 (75.0)
Anastomosis, n (%)	18 (75.0)
Preoperative treatment, n (%)^e^	
No treatment or mesalamine	6 (25.0)
Corticosteroids	3 (12.5)
Immunosuppressors (methotrexate, thiopurines)	14 (58.3)
Anti-TNF	11 (45.8)
Vedolizumab	1 (4.2)
Combo therapy immunosuppressors + biologics	9 (37.5)
Antibiotics	1 (4.2)
Postoperative treatment, n (%)^b^^,^^e^	
No treatment or mesalamine	5 (20.8)
Corticosteroids	0 (0.0)
Immunosuppressors (methotrexate, thiopurines)	8 (33.3)
Anti-TNF	10 (41.7)
Vedolizumab	1 (4.2)
Combo therapy immunosuppressors + biologics	4 (16.7)
Antibiotics	2 (8.3)
Presence of granulomata on histologic specimens, n (%)^c^	8 (33.3)
Delay of colonoscopy after index surgery (days), median [IQR]^f^	236.5 [167.0–278.8]
Rutgeerts score^f^	
i,0, n (%)	8 (33.3)
i,1, n (%)	2 (8.3)
i,2, n (%)	4 (16.7)
i,3, n (%)	1 (4.2)
i,4, n (%)	2 (8.3)
Clinical recurrence at 18 months, n (%)^c^	4 (16.7)

IQR, interquartile range.

a Two missing data.

b One missing data.

c Three missing data.

d Twelve missing data.

e Patients may have more than one treatment.

f Six missing data.

### Cell Culture

#### Enteric glial cells

Human myenteric glial cells were isolated from macroscopically healthy area of intestinal resections obtained from 13 control subjects (Table 3) who gave their informed consent to take part in this study. All the procedures were performed according to the guidelines of the French Ethics Committee for Research on Humans (DC-2008-402). Rodent glia were prepared from the colonic myenteric plexus of 12-week-old female Sprague-Dawley rats (Janvier Laboratories, Le Genest-St-Isle, France) according to the recommendations of the Animal Care and Use Committee of Nantes (France). Cultures of glia were characterized by immunocytochemistry and only used if 95% of the cells were GFAP^+^ and/or S100β^+^. Enriched primary cultures of myenteric glial cells were prepared^36^ and treated for 6–8 hours (RNA analyses) or 24 hours (flow cytometry, immunocytochemistry, T-cell adhesion) with 100 ng/mL LPS (Sigma-Aldrich, Saint-Quentin-Fallavier) or with 10 ng/mL IL-1β (PromoCell GmbH, Heidelberg, Germany) and 10 ng/mL TNFα (Sigma-Aldrich). The supernatants were carefully removed before the addition of T cells.

**Table 3:** Origin and Characteristics of the Human Myenteric Glia Used in Cell Culture

	Control
Culture number	12
Age, year, mean (min/max)	64 (16/93)
Sex: female/male	6/6
Cancer	8
Familial adenomatous polyposis	1
Stenosis	1
Diverticulitis	1
Polyps	1
Origin of the myenteric ganglia: colon/ileum	11/1

#### T lymphocytes

Rat T lymphocytes from fresh spleen or mesenteric ganglia were purified by negative cell sorting using anti-mouse immunoglobulin G coated Dynabeads (Thermo Fisher Scientific, Courtaboeuf, France).^37^ Human T lymphocytes were collected from the blood of healthy donors (Etablissement Français du Sang, Nantes, France).^38^ Briefly, after the removal of peripheral blood mononuclear cells (PBMC) on Ficoll, T lymphocytes were sorted using Pan T-cell isolation kit (Miltenyi Biotech, Paris, France). T-cell activation was performed using anti-CD28 (2.5 [rat] or 5 [human] μg/mL) and coated anti-CD3 (1 μg/mL) antibodies (BD-Bioscience). T lymphocytes were stained with 5 μmol/L of carboxyfluorescein-succinimidyl-ester (CFSE) (Thermo Fisher Scientific) before their addition to glia cultures.

#### Glia–T-cell co-cultures

Activated or non-activated T lymphocytes labeled with CFSE were added to the cultures of glia (ratio 1:1) and incubated for 2 hours. Then, the plates were carefully washed 5 times with phosphate-buffered saline (PBS) to remove the low adherent T cells and fixed with 4% paraformaldehyde in PBS.

#### ICAM-1/LFA-1 neutralization

To block the binding of ICAM-1 to LFA-1, activated T cells were added to the cultures of glia either after 1 hour of preincubation of glia with 10 μg/mL anti–ICAM-1 (BD Pharmingen) or isotype antibody (IgG1κ, Biolegend) or after 30 minutes of preincubation of T cells as well as glia with 0.1 mmol/L LG (SAR-1180, Sigma-Aldrich) dissolved in dimethyl sulfoxide and H2O (1:1 v/v).

#### Human dermal microvascular endothelial cells

Human dermal microvascular endothelial cells were purchased from Clonetics (Lonza, Heidelberg, Germany) and cultured in complete EGM-2MV medium (Lonza). Cells were treated for 24 hours with 100 U/mL of TNFα (R&D Systems) before fixation.

#### Rat macrophages

Fresh spleen cells were plated for 24 hours on 48-well plates. Non-adherent cells were removed by 3 washings. Adherent macrophages were characterized using Ox42 antibody. Cells were treated for 24 hours with 100 ng/mL LPS.

### Animal Studies

#### Winnie mice

Animal studies adhered to ARRIVE guidelines and standards. All experimental procedures were approved by the Victoria University Animal Experimentation Ethics Committee (AEETH: 17/016). Homozygous Winnie mice (Win/Win) with spontaneous chronic colitis resulting from a primary intestinal epithelial defect due to a point mutation in the *Muc2* mucin gene (C57BL/6 background)^39^ (5-week-old, n = 5, 14 ± 2 g; 6-week-old, n = 4, 18 ± 2 g; and 12-week-old, n = 52; 20 ± 4 g; males and females) and homozygous C57BL/6 mice generated from the heterozygous breeding (WT) (5-week-old, n = 4, 15 ± 2 g; 12-week-old, n = 27, 23 ± 4 g; males and females) were used in this study. No differences were observed in any parameters between heterozygous littermates and WT C57BL/6 mice^15^; therefore, WT C57BL/6 mice from the same breeding colony were used as healthy controls. All animals were obtained from the Victoria University Werribee Animal Facility (Melbourne, VIC, Australia) and housed at the Western Centre for Health, Research and Education Animal Facility, Sunshine Hospital (Melbourne, VIC, Australia). Mice were housed in OptiMouse cages (Animal Care Systems, Centennial, CO) with corn cob bedding 1/8-inch size (Corncobology, Mt Kuring Gai, NSW, Australia) enriched with shredded paper and cardboards on a 12-hour day/night cycle in a temperature-controlled (+22ºC) environment. All mice had free access to water and food, a standard fixed formulation diet for Laboratory Rats and Mice fortified with vitamins and minerals (SF00-100) (Specialty Feeds, Glen Forrest, WA, Australia). All efforts were made to reduce any animal suffering.

#### Treatment and analysis of the Winnie mice

After 1-week acclimatization period, 12-week-old Winnie (Win/Win) mice with active colitis were randomized into 2 groups and injected intraperitoneally twice a day with solvent (Sham, n = 9) or with 15 mg/kg LG (LG, n = 14) dissolved in 2% Cremofor, 2% ethanol, 96% sterile water. At day14, mice were euthanized by cervical dislocation or cardiac puncture under anesthesia, and tissues were immediately collected. Disease progression was evaluated using the disease activity index, which combines scores for weight loss, stool consistency, rectal symptoms, and the colon weight/length ratio.^40^ The concentration of lipocalin-2 (Lcn-2) was quantified noninvasively in day 14 fecal supernatants using the Lcn-2 ELISA kit (R&D Systems, Minneapolis, MN).^41^ The morphology of the distal colon was investigated for each animal in 20-μm cryostat sections stained with hematoxylin-eosin.^15^ Colonic crypt architecture, epithelium damage and ulceration, mucosa and muscle thickness, smooth muscle dysplasia, and leukocyte infiltration were assessed using images taken by Zeiss Axio Imager microscope (Zeiss, Marly le roy, France).

#### Motility

Ex vivo colonic motility experiments were performed as described previously.^42^^,^^43^ Briefly, pellet-free colons were positioned horizontally and cannulated at the oral and anal ends in an organ bath superfused with Kreb’s solution at 37°C. Height of the reservoir was adjusted to maintain the intraluminal pressure (0 to +2 cm H_2_O), while the anal end was coupled to an outflow tube with a maximum backpressure of 2 cm H_2_O. After 30 minutes of equilibration, contractile activity was recorded (2 sequential 30-minute periods) by a video camera positioned above the organ bath. Videos were transposed to spatiotemporal maps with Scribble v2.0 and analyzed using the MATLAB v2017a software to assess colonic motility parameters.^44^

### Immunochemistry

All the antibodies used in this study (Table 4) were first tested on cell cultures, whole-mount tissues, and/or tissue sections containing cells expressing the molecule of interest in the species concerned. Antibodies were validated by verifying that they stained only the expected cells (positive control). In each experiment, sister cell cultures or sections were labeled without primary antibody to avoid false-positive staining (negative control). Bibliographic references were added for the antibodies routinely used in our laboratory. Some of them are used in clinical practice for diagnostic purposes as mentioned in Table 4.

**Table 4:** List of Antibodies Used in the Study

Target	Clone	Use Reactivity	Isotype	Host	Company – reference (dilution)
CD3^a^^,^^18^	F7.2.38	IHCp, ICC Human	IgG1, κ	Mouse	Dako Agilent - M7254 (1/100)
CD3	Polyclonal	IHCp Human	/	Rabbit	Dako Agilent – GA503 (1/2)
CD3^19^	G4.18	Activation Rat	IgG3, k	Mouse	BD Bioscience - BD 554829 (1/1000)
CD3	HIT3a	Activation Human	IgG2a, k	Mouse	BD Bioscience - BD 555336 (1/1000)
CD3	17 A2	IHC Mouse	IgG2b, κ	Rat	BD Bioscience - BD 555273 (1/200)
CD4^a^	4B12	IHCp, ICC Human	IgG1	Mouse	Novocastra NCL-L-CD4-368 (1/50)
CD8^a^	C8/144B	IHCp, ICC Human	IgG1	Mouse	Dako Agilent – IS623 (1/2)
CD11b/c^19^	OX42	Purif, ICC Rat	IgG2a, κ	Mouse	BD Bioscience - BD 550299 (1/125)
CD28^19^	JJ319	Activation Rat	IgG1	Mouse	BD Bioscience - BD 554993 (1/400)
CD28	CD28.2	Activation Human	IgG1	Mouse	BD Bioscience - BD 555725 (1/200)
CD45R^19^	HIS24	Purif Rat	IgG2b, κ	Mouse	BD Bioscience - BD 554879 (1/500)
CD45RA	4KB5	IHCp, ICC Human	IgG1, κ	Mouse	Invitrogen – MA5-12490 (1/200)
NKR-P1A	3.2.3	Purif Rat	IgG1, κ	Mouse	Invitrogen - MANK07 (1/700)
Hu^18^	Polyclonal	IHCp Human	/	Rabbit	Santa Cruz Biotechnology -sc-5977 (1/500)
Hu^20^	Polyclonal	IHCp Mouse	/	Human	Gift CHU Nantes (1/500)
ICAM-1	BBIG-I1 (11C81)	IHC, Human	IgG1	Mouse	Bio -Techne – BBA3 (1/200)
ICAM-1 FITC	HA58	FC Human	IgG1, κ	Mouse	Biolegend 353108 (1/200)
ICAM-1	1A 29	ICC, FC, Neu Rat	IgG1, κ	Mouse	BD Bioscience - 554967 (ICC,1/200; FC, 1/200; Neu 1/100)
ICAM-1	YN1/1.7.4	Mouse	IgG2b, κ	Rat	Abcam - ab119871 (1/200)
S100β^a^^,^^18^^,^^21^	Polyclonal	IHCp, ICC Human, Rat	/	Rabbit	Dako Agilent - GA504 (1/2 to 1/3)
S100β	Polyclonal	IHCp Human	/	Guinea pig	Synaptic Systems – 287 004 (1/500)
S100β	Polyclonal	IHC Mouse	/	Rabbit	Abcam - ab52642 (1/500)
TCRα/β^22^	R73	ICC Rat	IgG1	Mouse	BD Bioscience - BD 554911 (1/200)
Mouse IgG (FITC)	Polyclonal	IHC, ICC	/	Donkey	Jackson Immunoresearch 715-095-150 (1/200)
Rabbit IgG (Cy3)	Polyclonal	IHC, ICC	/	Donkey	Jackson Immunoresearch 711-165-152 (1/500)
Human IgG (AF647)	Polyclonal	IHC, ICC	/	Donkey	Jackson Immunoresearch 709-605-149 (1/500)
Rat IgG (Cy3)	Polyclonal	IHC, ICC	/	Donkey	Jackson Immunoresearch 712-165-150 (1/500)
Rabbit IgG (FP488)	Polyclonal	IHC, ICC	/	Donkey	Interchim FP-SA5110 (1/200)
Rat IgG (Cy5)	Polyclonal	IHC, ICC	/	Donkey	Jackson Immunoresearch 712-175-150 (1/500)
Mouse IgG (Cy3)	Polyclonal	IHC, ICC	/	Donkey	Jackson Immunoresearch 715-165-151 (1/500)
Mouse IgG (Cy5)	Polyclonal	IHC, ICC	/	Donkey	Jackson Immunoresearch 715-175-151 (1/500)
Rabbit IgG (Cy5)	Polyclonal	IHC, ICC	/	Donkey	Jackson Immunoresearch 111-175-144 (1/500)
Guinea pig IgG (AF488)	Polyclonal	IHC, ICC	/	Goat	Invitrogen A11073 (1/400)
IgG1	MOPC-21	Neu	IgG1, κ	Mouse	Biolegend 400101 (1/200)

a antibody used for diagnosis. FC, Flow Cytometry; ICC, Immunocytochemistry; IHC, Immunohistochemistry, IHCp, IHC paraffin; Neu, Neutralization.

Human 5-μm full-thickness sections were deparaffined using standard procedures and incubated in antigen-blocking solution (Dako Agilent, Santa Clara, CA) for 1 hour at room temperature. LMMP whole-mount preparations were isolated from the mouse distal colon (1 cm^2^), fixed overnight with Zamboni’s fixative (2% paraformaldehyde, 0.2% picric acid) at 4°C, and incubated for 1 hour in 10% donkey serum (Merck Millipore, Bayswater, VIC, Australia) diluted in PBS–Triton 0.1%. Cell cultures were fixed with 4% PAF and incubated for 2 hours at room temperature in PBS supplemented with 5% (vol/vol) horse serum (Thermo Fisher Scientific) and 0.25% (vol/vol) Triton X-100. Primary antibodies (Table 4) were diluted in Dako diluent (paraffin tissue sections) (Dako Agilent) or in PBS-5% horse serum. Tissues or cells were incubated overnight at 4°C with primary antibodies, washed, and incubated for 2 hours with secondary antibodies. After counterstaining with DAPI (1/1000, Sigma-Aldrich), slices or cells were mounted in the ProLong Gold Antifade Mountant (Thermo Fisher Scientific) or in the Dako mounting media (Dako Agilent). As negative control, the same immunostaining protocol was performed without the primary antibody. Photomicrographs were acquired using the stereo zoom microscope Axio Zoom.V16 (Zeiss, Oberkochen, Germany) coupled to apotome or confocal microscope Nikon A1 RSi (Nikon SAS, Champigny sur Marne, France) or INCell2200 (GE Healthcare, Velizy-Villacoublay, France).

### Cell Counting and Micrograph Analyses

#### Analysis of the human transmural 5-μm sections

Analyses were performed on micrographs acquired by a Zeiss Axio Imager microscope (Zeiss, Marly-le-Roy, France). The entire length of the interface between circular and longitudinal muscle cell layers was visualized, and images were acquired when ganglia ENS staining was present. T cells were counted when they were either inside or at the border of the ganglia and apposed to ENS staining. Apposition was defined as overlapping or strict contact between neural and T-cell staining (no black pixel between the two staining; pixel size, 0.413 μm). T-cell apposition to enteric glia or neurons was expressed in mean number of T cells per ganglia or per cells, respectively. Counting was performed blindly; sociodemographic, clinical, histologic, and endoscopic data of patients were collected after counting.

#### Analysis of the co-cultures

T cells adherent to enteric glia were automatically counted using the IN Cell Analyzer Developer tool. In total, 81 micrographs of each well, corresponding to 36 mm^2^ of the culture, were acquired using the IN Cell Analyser 2200 (GE Healthcare) and analyzed using the INCell Developer Toolbox 1.9.3. T cells were defined as DAPI^+^CFSE^+^ cells with a size ≥50 μm, whereas enteric glial cells was identified as S100β^+^cells. T cells were considered as adherent when at least 5% of the CFSE^+^ and S100β^+^ staining was overlapping, even if T cells did not adhere to the culture plastic plate. For each condition and experiment, analyses of interactions were performed on 3 independent wells, and the median value was used in comparison studies.

#### Analysis of the whole-mount mice tissues

The number of CD3^+^DAPI^+^ cells apposed (as defined for human sections) to S100β^+^ glia inside myenteric ganglia were counted from confocal images acquired with the Nikon Eclipse Ti multichannel confocal laser scanning system (Nikon, Japan). Myenteric ganglia were randomly selected for a total of 12 ganglia per animal and analyzed using compressed micrographs. Data are expressed as the number of T cells per ganglia.

### Flow Cytometry

After 24 hours of treatment, enteric glial cells were detached by Accutase enzyme (STEMCELL Technologies SARL, Saint-Egreve, France) and immunostained for 30 minutes on ice with ICAM-1-FITC antibody (Biolegend, San Diego, CA) (human cells) or with ICAM-1 antibody (BD, 554967) and then with Alexa Fluor 488 donkey anti-mouse immunoglobulin G (H+L) antibody (TF, A21202) (rat cells). Analyses were performed using BD FacSymphony A5 with BD FACS Diva software version 8.0. Data and graphs were analyzed and generated using FlowJo v10.8 Software (BD Life Sciences).

### Quantitative Polymerase Chain Reaction Analyses

Total mRNAs were prepared according to the Macherey-Nagel manufacturing’s procedure, and reverse transcription was performed using the Superscript III reserve transcriptase (Thermo Fisher Scientific). Real-time quantitative polymerase chain reaction was carried out using SYBR Green assays and run on the StepOnePlus system (Thermo Fisher Scientific). Forward and reverse primers for neural cell adhesion molecule and ICAM-1 are referenced in Table 5. Gene expression was normalized to the RPS6 reference gene.

**Table 5:** Table of Forward and Reverse Primers

Target	Primers	
h-ICAM-1	Up	ATGGCAACGACTCCTTCTCG
	lp	GCCGGAAAGCTGTAGATGGT
r-ICAM-1	up	CGGACTTTCGATCTTCCGACTA
	lp	TTTGTGCTCTCCAGGGTCAG
	lp	CTTGAACTCAGTGGCTGCAC
h-RPS6	up	AAGCACCCAAGATTCAGCGT
	lp	TAGCCTCCTTCATTCTCTTGGC
r-RPS6	up	GCATTGTGGATGCCAACCTG
	lp	GTCCTGGGCTTCTTACCTTCTT

### Single-Cell Data Analysis

Single-cell data reported in Kong et al^13^ were downloaded from the Broad Single Cell Portal (SCP1884). Stromal compartments from colon and terminal ileum samples of healthy individuals and patients with Crohn’s disease at varying levels of inflammation were separately analyzed using Seurat (4.4.0) and bioconductor DESeq2 (1.40.2). In both cases, raw counts were aggregated to the sample and cell type levels using Seurat’s AggregateExpression () function. DESeq2 was specifically run on glial cells aggregated counts to compare gene expression according to the patient’s health status.

### Statistical Analyses

Statistical analyses were performed using GraphPad Prism (GraphPad Prism 9, GraphPad Software Inc, San Diego, CA). ∗*P* < .05, ∗∗*P* < .01, ∗∗∗*P* < .001, ∗∗∗∗*P* < .0001.
